# The “Autothixotropic” Phenomenon of Water and its Role in Proton Transfer

**DOI:** 10.3390/ijms12117481

**Published:** 2011-10-31

**Authors:** Nada Verdel, Igor Jerman, Peter Bukovec

**Affiliations:** 1 Institute Bion, Stegne 21, 1000 Ljubljana, Slovenia; E-Mail: igor.jerman@bion.si; 2 Department of Inorganic Chemistry, Faculty of Chemistry and Chemical Technology, University of Ljubljana, Aškerčeva 5, 1000 Ljubljana, Slovenia; E-Mail: peter.bukovec@fkkt.uni-lj.si

**Keywords:** conductivity, hydrophilic surfaces, ions, thixotropy, water

## Abstract

In an experimental study, significantly higher conductivity values than those of freshly prepared chemically analogous solutions were found in aged (~one year old) aqueous solutions, except for those stored frozen. The results surprisingly resemble a previously noticed phenomenon in liquid water, which develops when water is stored in closed vessels. This was observed as a disturbing phenomenon in gravimetric measurements and in luminescence spectroscopy measurements. The phenomenon was termed “autothixotropy of water” due to the weak gel-like behavior which develops spontaneously over time, in which ions seem to play an important role. Here, according to experimental results we propose that contact with hydrophilic surfaces also plays an important role. The role of the “autothixotropy of water” in proton transfer is also discussed.

## 1. Introduction

Water is an unusual compound, yet not entirely different from other liquids. It is generally agreed that the hydrogen bonds that connect water molecules are responsible for its anomalies [1]. Its extraordinary properties are critical in the establishment and maintenance of life [2,3]. Hence, what we may ask is what role water plays in cells, the integral parts of living organisms. According to conventional views, water is nothing but an unproblematic (ordinary) solvent for life-sustaining molecules like proteins, DNA and RNA. In contrast, according to Bizzarri and Cannistraro, the dynamics of the protein and solvent are so strongly coupled that they “should be considered as a single entity with a unique rough energy landscape” [4].

**Figure 1:**
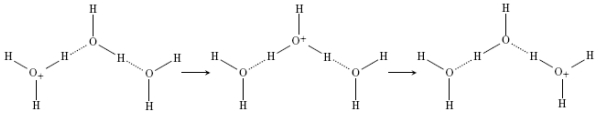
The transport of hydrogen ions (H^+^) through water is accomplished by the Grotthuss mechanism, in which hydrogen bonds (dashed lines) and covalent bonds (solid lines) between water molecules are broken and re-formed.

Generally it is thought that the impact of surfaces on the hydrogen bonded network of water extends to a distance of no more than a few molecular layers. Yet Zheng and Pollack [5] found that colloidal and molecular solutes are excluded from the vicinity of various hydrophilic surfaces. The depth of these solute-free exclusion zones (EZ) amounts to a few hundred μm, EZs are physically distinct and less mobile than the bulk [6]. According to Guckenberger and coworkers [7], the thin water layer next to a hydrophilic surface exhibits a surprisingly increased conductivity, higher than that of bulk water by up to five orders of magnitude. The water film on the surface of a hydrophilic insulator can be considered to function as a conductive coating. They ascribed the high conductivity to a proton hopping mechanism along water structured at surfaces (see Figure 1). Sasaki [8] found similar results for the conductivity of collagen. Namely, the conductivity of collagen, the most abundant protein in mammals, depends remarkably on the amount of hydration water.

In a previous study [9,10] we continued the work of Elia [11]. Elia and coworkers explored the physico-chemical properties of aqueous solutions of NaHCO_3_ treated mechanically by iterated dilution and succussion (vigorous shaking). They repeated the processes to extreme dilution, where the chemical composition of the end solution was identical to that of the solvent. They measured the electrical conductivities of aged, extremely dilute solutions treated mechanically by vigorous shaking, and compared the results with electrical conductivity values of one day old untreated analogous solutions [11–16]. They noticed significantly higher electrical conductivities in aged mechanically treated solutions than in analogous fresh untreated solutions. They attributed their unusual results to water’s self-organizing abilities triggered by the input of kinetic energy during succussion which promotes the ordering of water molecules. Namely, larger water clusters accelerate the Grotthuss mechanism of proton transfer that predominates in aqueous hydrogen carbonate solutions [17]. According to this mechanism an ‘excess’ proton or protonic defect diffuses through the network of hydrogen-bonded water molecules through the formation or cleavage of hydrogen and covalent bonds (see Figure 1) [18].

We [9,10] found that the first day after mechanical treatment (vigorous shaking) the conductivity values of solutions did not differ from the analogous untreated solutions. Accordingly we followed the work of Elia [11] and aged the treated and untreated aqueous NaHCO_3_ solutions. In contrast to the results of Elia [11], no differences in conductivities of aged mechanically treated solutions and aged untreated solutions were found [9,10]. Yet all aged solutions (treated and untreated) had significantly higher conductivity values in comparison to the conductivity of chemically analogous fresh solutions and no excess conductivity was found in frozen samples. This is surprisingly similar to the observations of Vybíral and Voráček [19] who noticed that liquid distilled water, left to stand undisturbed for some time in closed vessels, spontaneously develops “autothixotropic” properties where ions seem to play an important role. Thixotropy is a property of certain gels and liquids that are viscous under normal conditions, but flow (or become less viscous) when shaken or otherwise stressed.

In what follows, we review the possible formation of a structured network of hydrogen bonds between water molecules and ions in aqueous solutions that are left to stand undisturbed for some time, leading to the development of autothixotropic properties where hydrophilic surfaces also play an important role. We propose the autothixotropy to be the cause of the faster proton transfer in aged solutions along the lines of the Grotthuss mechanism, in comparison to proton transfer in freshly prepared solutions with identical chemical composition.

## 2. Discussion

### 2.1. Water as Liquid and Solvent

There are few molecules structurally simpler than water, yet the behavior of water is very complex. The sp^3^ hybridized oxygen atom creates an essentially tetrahedral coordination geometry. The oxygen atom contains four tetrahedrally arranged electron pairs. Two are occupied by covalent bonds between hydrogen and oxygen, whereas two are free, directed to the corners of the tetrahedron [20]. Tetrahedral coordination enables each molecule of water to coordinate with four other water molecules. Since the molecules are mobile in the liquid phase, the four hydrogen-bonding sites are not necessarily fully occupied. Neutron-scattering measurements of radial distribution functions and computer simulations suggested that at room temperature, the number of hydrogen-bonded nearest neighbors for water molecules averages about 3.5 [21,22].

#### 2.1.1. Hydrogen Bonds

In water, the long-range attractive forces of hydrogen bonds predominate. Molecules in water are less densely distributed than are the particles of a simple liquid, in which the particles experience almost no intermolecular force until they touch. In water the particles are held apart by hydrogen bonds, which impose geometrical constraints on the molecular positions: hydrogen bonds are linear along the axis between the two oxygen atoms. If the axis is bent, the hydrogen and oxygen orbital overlap is poorer and consequently the H-bond is weaker [21–23].

One of the most important features of hydrogen bonds are their cooperative effects: namely, formation or cleavage of one facilitates the formation or cleavage of the next hydrogen bond in line. The second important feature of H-bonds is their specificity: hydrogen bonds only form between hydrogen donors (H) and acceptors (e.g., O or N). The hydrogen bonded network of water is internally differentiated by the participation of individual water molecules in the hydrogen bonds; the molecules occur: (a) either as proton donors that donate both of their hydrogen atoms; or (b) as proton acceptors that accept two hydrogen atoms; or (c) as a combination of the two states. When molecules act as both donor and acceptor at the same time, the network of hydrogen bonds is established. In the network hydrogen and oxygen atoms are connected only in fixed positions and at fixed angles. Hence, hydrogen bonds have a spatial character, wherein they differ essentially from other forces between neutral molecules [22,24].

In pure liquid water hydrogen bonds have an average lifetime of 1 ps, so although there exists an extended, infinite dynamic three-dimensional network, there are no long-lived “ice-like” structures. Broken hydrogen bonds re-form almost immediately, in 200 femtoseconds [25]. If we take into consideration water’s role as a solvent—for example within the cell—a fundamental question arises; how is the lifetime of hydrogen bonds affected by a solute molecule or the proximity to a surface. In any case, the structural response of liquid water depends on whether the foreign body is hydrophobic or hydrophilic. However, in more or less all cases the issue is debatable and is still not well resolved [23].

#### 2.1.2. Influence of Ions

Concerning the effects of ions on the bulk properties of liquid water, such as viscosity, it has been suggested that ions alter water’s hydrogen-bonding network [26]. This appears to be confirmed by, for example, neutron diffraction experiments [27]. Whereas according to Krekeler and Delle Site local polarization effects, induced by mono and divalent positive ions in water should extend to the first hydration shell only, the water-water interaction plays a dominant role even in the first shell, independently of the size or charge of the ion [28]. Furthermore, using femtosecond pump-probe spectroscopy Omta and coworkers found that ions have no influence on the rotational dynamics of water molecules outside the first solvation shells of the ions [29]. Their results demonstrate that the presence of ions does not lead to growth or breakdown of the hydrogen-bonded network in liquid water.

Turton and coworkers separated the effects of rotational and translational motions of the water molecules by the ultra-fast Kerr effect and dielectric relaxation spectroscopic measurements [30]. The results of these two methods showed that salt solutions behave like a supercooled liquid approaching a glass transition, where rotational and translational motions become decoupled. At a concentration of, for example, 4.4 mol/L MgCl_2_, there are overall approximately eleven water molecules per cation, approximately seven of which form a stable hydration shell around the cation in which rotation is strongly impeded. The remaining four water molecules are assumed to form small “pools”, where rotation is still as free as in the bulk, but translations are slowed down. At the glass transition concentration the density of clusters is so great that they jam, effectively turning the electrolyte solution into a glass. The hydrophilic particles (ions) somehow trap the translational motion of water molecules. And what is the influence of hydrophilic surfaces, we may ask.

#### 2.1.3. Influence of Hydrophilic Surfaces

Cheng and coworkers found that superficial water molecules are preferentially oriented towards a hydrophilic surface and that they are more weakly hydrogen-bonded than in the bulk [31], which was confirmed by Jena and Hore [32]. The latter [32] found that as the electrolyte concentration increases, the water molecules nearer to the surface are better aligned and that the width of the hydration shell is thinner. However, it cannot be expected that water adjacent to a hydrophilic surface would be bulklike: even simple liquids are layered by packing effects [23,33]. Both the densification induced by molecular packing and the lateral ordering due to surface structure would be expected to disrupt the hydrogen bonding in the water layer. So, the question arises do the differences go beyond those we might expect from the theory of structurally simpler liquids [23].

The recent findings of Pollack and coworkers could indicate that next to hydrophilic gels a fourth aggregate state of water exists, namely a liquid crystalline phase. The research of Pollack *et al*. [5,6,34–38] may indicate that water molecules are ordered in some few hundred thousand layers away from hydrophilic gels. Under the microscope Zheng and Pollack [5] found that water forms massive exclusion zones (EZs) completely (or almost completely) without solutes next to the surfaces of hydrophilic gels. The clear zone next to the gel surface was a few hundred microns deep and stayed stable for days and even weeks.

In searching for possible errors in their findings Pollack and coworkers found that EZs could represent a common phenomenon in water which was already noticed more than fifty years ago. For example, Henniker in his review article of 1949 points out numerous experimental reports showing impressive long-range ordering of various liquids, including water [39]. Pollack’s group continued their research and found EZs present near many different hydrophilic gels: polyvinyl alcohol gel, polyacrylamide gel, gel from polyacrylic acid, ionomer Nafion and biological tissues like collagen. Exclusion was proposed to exist even in the vicinity of a hydrophilic monolayer, whereas no exclusion was found in the vicinity of hydrophilic surfaces without functional groups, such as for example stainless steel wire. Therefore, they proposed that EZs only form next to hydrophilic surfaces that contain functional groups able to form H-bonds with water. Excluded solutes included microspheres, erythrocytes, bacteria, colloidal gold and molecules like serum albumin, marked with fluorescent dye, and a fluorescent dye with a molecular weight of 200–300 Da [6].

NMR and IR images indicate that EZ water has a lower mobility and is more ordered than water in the bulk [6]. pH sensitive dyes show an unusually low pH of bulk water next to an EZ, which indicates a high concentration of protons due to high ionization of water. Additionally, measurements of electrical potential showed that an EZ is negatively charged in comparison to the bulk, which indicates a high concentration of hydroxyl ions in the EZ. Furthermore, radiation by visible and particularly IR light is considered to deepen the width of the EZ. According to these results, Voeikov and Del Giudice [40] proposed that the electrons in EZ water are less bound than in bulk water, which is related to the complex behavior of water and to its dynamic properties.

### 2.2. Time Related Changes in Aqueous Solutions

Time related changes in the properties of aqueous solutions are crucial in the interpretation of our results [9,10] as the excess conductivity found in comparison to the conductivity of fresh chemically analogous solutions was found only after a period of ageing. No correlation with previous input of kinetic energy (iteration of dilution and succussion) was found, whereas an increase in the concentration of ions, as well as in the ratio between the hydrophilic glass surface and solution volume, increased the conductivity values. No excess conductivity was found in samples stored frozen. We [9,10] attributed these results to the self-organizing abilities of liquid water which according to Vybíral [41] becomes autothixotropic when left to stand undisturbed.

Vybíral and Voráček observed a disturbing phenomenon during their gravimetric measurements [19]. The phenomenon was relatively weak and appeared only on a macroscopic scale when water was left standing for some time. This resulted in a force of mechanical resistance against an immersed body when changing its position. They used both static and dynamic macroscopic methods. In the static method the moment of force necessary for a significant turn of a stainless steel plate, hung on a thin filament and immersed in aged water, was measured. By a given angular torsion of the filament in the aged water a certain moment of force was reached that was accompanied by an impressive change in the angular position of the plate. In the dynamic methods, both oscillation of the plate, as well as a very slow fall of a small ball in aged water were observed. They called the phenomenon “autothixotropy of water” and proposed an explanation involving cluster formation by H_2_O molecules in aged water. As the phenomenon did not appear in deionized water, a preliminary conclusion was drawn that the phenomenon is determined by the presence of ions.

Similar time related changes in the structure of water were also noticed by Lobyshev and coworkers [42]. They found that distilled water possesses weak luminescence in the near UV and visible regions of the electromagnetic spectrum. Its excitation spectrum is complex and has two maxima at 260 and 310 nm. The corresponding emission spectra, apart from narrow lines due to Raman scattering, are represented by wide bands at 360 and 410 nm that depend on the unusual properties of water. Namely, the intensity of luminescence depends on the duration of water storage in a closed vessel and on the addition of trace amounts of both luminescent and non-luminescent substances. After a week’s storage of the samples in the dark, the intensity of the emission spectrum bands increased by about 20% and after two to four months it showed a two fold increase. Further storage of the samples did not change the intensity appreciably. They noted that the electrical conductivity of water in different samples varied from 0.08 μs/cm to 3.9 μs/cm, but the value of the conductivity did not correlate with the intensity of luminescence.

To clarify the possible role of dissolved gases, the samples of water stored for a long time were degassed with a water-jet pump, until bubbles were no longer released on agitation. The spectra of the degassed sample did not differ from control samples. To check if the origin of the modified properties of water was the modification of the water structure, a minimal content of luminescent and non-luminescent additives was added. In dilute solutions in which one may ignore the effects of concentration quenching and reabsorption, the intensity of luminescence should depend linearly on the concentration of the luminescent additives and be independent of time. Addition of a non-luminescent additive should not lead to an increase in the luminescence intensity. However, in aqueous solutions containing 1 μmol/L of a non-luminescent dipeptide, a twofold increase of luminescence intensity was noticed compared to the luminescence of pure water without any change in the position of the maxima. Therefore, the observed phenomenon could not be reduced to the luminescence of admixtures in water, but was attributed to the unique properties of water, its dynamic structure and polymorphism. Lobyshev and coworkers came to the conclusion that water and aqueous solutions should be regarded as continuous polymorphous self-organizing systems [42].

### 2.3. The Possible Role of the “Autothixotropic” Properties of Water on Living Beings and Proton Transfer

In the last two decades it became clear that water is not only the solvent of biological molecules, like proteins, RNA and DNA, but actively participates in reactions essential for the establishment and maintenance of life. Through its unusual and unique properties it influences the modifications of conformational states of proteins, the creation of two-layer phospholipid membranes and the recognition sites of DNA and RNA. In the 16th century Paracelsus wrote that “water was the matrix of the world and of all its creatures” [43]. In contrast, until very recently, molecular biologists held a completely different picture of the role of water. In spite of acknowledging that liquid water has some unusual and important physical and chemical properties, biologists have regarded water only as a passive backdrop on which life’s important molecules are arrayed [23]. It used to be a common practice to perform computer simulations of biomolecules in a vacuum, which reflected the prevailing notion that water does little more than moderate the basic physicochemical interactions responsible for molecular biology [44]. Thus, we should ask what is the part that water plays in biochemical processes in the cell?

According to Frauenfelder and coworkers [45], the active volume of proteins extends beyond their physical boundaries and depends on their interactions with vicinal water, *i.e.*, their hydration shells. The structure and dynamics of the hydration shells determines the biological function of proteins. Or put differently, the hydration shell of a protein and the protein are two different, yet strongly interconnected parts. Many proteins react indirectly with other proteins or molecular substrates through water molecules in the hydration shell [46].

Many proteins locate their target sequence by first non-specifically binding to DNA, then by linearly diffusing or hopping along the DNA until either the protein dissociates from the DNA or finds its recognition sequence. The diffusion of a nonspecifically bound protein along the DNA is (*in vitro*) 2000-fold slower than the diffusion of a free protein in solution [47]. Rau and Sidorova [47] attribute a factor of 40–50 to rotational friction resulting from following the helical path along the DNA backbone, while following the DNA helix protein-binding site is directed towards DNA [48]. The remaining part of the diffusion velocity reduction can be ascribed to two possibilities: transiently broken electrostatic interactions between the DNA and the protein, or disruption of the water structure at the protein-DNA interface [47].

The result of a Monte Carlo computer simulation by Fuxreiter and coworkers suggested that the protein-DNA interfacial water could serve as a “hydration fingerprint” of a DNA sequence [49]. Similarly, the contact of a hexapeptide with a titania surface takes place via a pair of oppositely charged groups on the peptide that at the outset recognize the water layers at the interface, not the titania surface (TiO_2_) itself, and only then are adsorbed on its surface [50]. Namely, the water molecules orient according to the electrostatic interactions of the TiO_2_ surface and therefore attract oppositely charged functional groups on the peptide. Likewise, again, two layers of structured water form on the surface of hydroxylated quartz. The structured water molecules mediate the binding of amino acid residues onto the quartz surface via electrostatic interactions [51]. Samsonov and coworkers found that water molecules can play an important role in interaction conservation at protein interfaces by allowing sequence variability in the corresponding binding partner [52]. By its role as a mediator, water provides mobility to proteins which would be considerably diminished via direct protein-substrate interactions.

In 2004 a breakthrough in research on water structure occurred, when Naguib and coworkers trapped water in 2–5-nm-diameter channels of closed multiwalled carbon nanotubes (MWCN) [53]. The pictures they recorded through transmission electron microscopy revealed disordered gas/liquid interfaces contrasting with the smooth curved menisci visualized previously in MWCN above 10 nm. They also noticed that the mobility of water under the constrained conditions was greatly retarded compared to unconstrained water. The molecules of water interacted only among each other, and therefore an empty space occurred between liquid water and the nanotube wall. This discovery shows that water in confined spaces is deeply structured, which gives rise to an important question; namely what is the proton transfer rate in such ordered water?

Curiously, hydrogen cations diffuse faster through water than do other cations. In fact, their diffusion in water is 4.5 times faster than of any other cation. Their transfer involves the concerted making and breaking of many bonds in hydrogen bond network of water molecules, in a process known as the Grotthuss mechanism [54–56] (see Figure 1). Hydrogen cations in water are hydrated: they exist either as complex with individual water molecules (H_3_O^+^), forming Eigen ions (H_9_O_4_ ^+^) [57], or are shared equally by two water molecules to form Zundel ions (H_5_O_2_ ^+^) [58]. The exact form of the hydrated hydrogen ions has long received much attention [55,59]. Agmon and coworkers proposed that the molecular mechanism consists by isomerization between two protonated water cations: the more stable Eigen complex and the transition structure, the Zundel cation [55,60,61]. This was statistically proven by molecular simulations of Markovitch and coworkers [61].

Markovitch and coworkers [61] proposed that the proton conduction in water is, in agreement with Eigen [57], rather diffusive. It does not involve correlated coherent hopping over long hydrogen bonded water chains as previously anticipated. However, this may change when a proton hops between a donor and an acceptor like for example, in acid-base reactions. Recently, by using time-resolved IR spectroscopy measurements long-ranged proton transfer between acid-base intervening water molecules was found [62,63]. Likewise, computational simulations [64,65] and terahertz time-domain spectroscopy measurements [66] showed that large water molecule clusters are involved in the mechanism underlying proton mobility. Lapid and coworkers [65] suggest that proton mobility is a cooperative phenomenon. Hence, the proton conduction is proposed to be faster when the water molecules are more ordered [17,18]. Further, according to Ling [67] and Pollack [6], water molecules in the vicinity of membranes and surfaces of proteins are ordered in “macroscopic” dimensions.

**Figure 2:**
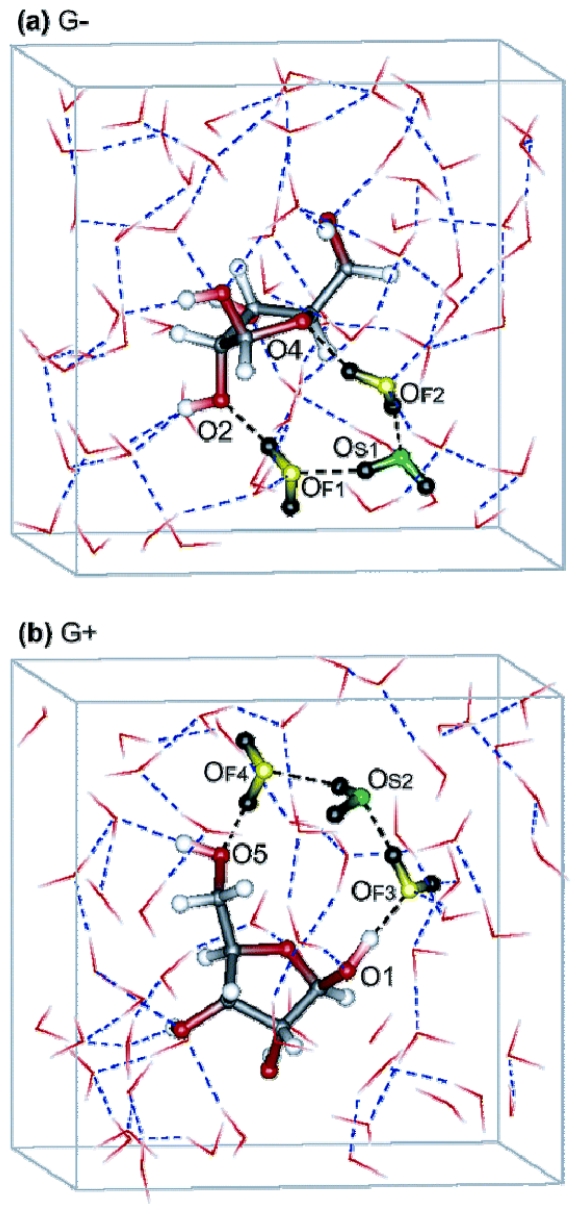
Representative configurations showing circular hydrogen bond networks near the ring oxygen in hydrated G− (**a**) and G+ (**b**) rotamers of β-ribofuranose sampled from ab initio MD trajectories by Suzuki and Soto [69]. Hydrogen bonds in the circular hydrogen bond networks are colored separately in black, while others are in blue. The three water molecules that form the circular hydrogen bond networks are represented by ball-andsticks, where oxygens of the first and second hydration shell water molecules are colored separately in yellow and green with hydrogens in black, as well as labeled by the subscripts F and S, respectively. β-ribofuranose and the other water molecules are represented by ball-and-sticks and sticks, respectively (carbon atoms, grey; oxygens, red; hydrogens, white).

According to the biochemistry textbooks, living organisms are charged predominantly by accumulating hydrogen ions on one side of a membrane, and discharged by hydrogen cations flowing back to the other side. Hydrogen ions are transferred across the biological membranes by special membrane proteins called “proton pumps”. It is presumed that the basic parts of proton pumps are structured (or organized) chains of water molecules [68–70]. Using molecular dynamics simulations, Suzuki and Sota found loops of hydrogen-bonded water molecules between hydroxyl groups at the surface of the sugar β-ribofuranose (see Figure 2) [69]. Such “circular hydrogen bonded networks”, first identified in crystal structures by Saenger [68] and persisting at least for several picoseconds in solution, may increase the dipole moments of the water molecules concerned, and could also act as proton-conducting pathways.

Proton-hopping in the hydrogen-bonded chain remains a viable process for water molecules in confined geometries in which the formation of a bulk-like, three-dimensional network is not possible. The existence of such “proton wires” has been reported in a variety of proteins, where they provide proton-conduction channels connecting the interior and exterior of the molecules. For example, there is a 23 Å water wire in the photosynthetic reaction centre of *Rhodobacter sphaeroides*, disruption of which disturbs the functioning of the protein complex [70].

The results of our study [9,10] showed that the excess conductivities found could be proportional to the ratio between the hydrophilic surface and the volume of the solution. If the dimensions we used were extrapolated to the ratios in the cell, it could probably be confirmed that the protons in the ordered water of proton pumps are, in accordance with Ho [71], “super”-conductive.

## 3. Conclusions

Water plays a wide variety of roles in the chemistry of life. Our purpose was to review the possibility of the following assumption: with time water molecules self-organize into clusters as a consequence of water’s self-organizing ability; ions and hydrophilic surfaces may play a significant role in the stability of these clusters. We suggest that this phenomenon is already used by organisms, for example, by special membrane proteins called “proton pumps”.

Ions seem to slow down the translational motions of water molecules and hydrophilic surfaces may order water molecules over much larger distances than previously expected; translational motion refers to motion that changes the position of a molecule without rotation. The time related changes in water’s physicochemical properties were indeed noticed in conductivity measurements as an increase of conductivity, irrespective of the chemical content, in luminescence spectroscopy as an increase in the intensity of the bands in water’s emission spectrum, or in dynamic gravimetric measurements as the very slow fall of a small ball in aged water. These findings point in the same direction—the autothixotropic properties of water, developed spontaneously with time. However, these studies were totally unrelated, in most cases authors were searching for other properties. Hence, we propose it is high time that the autothixotropic or gel-like behavior in water be clarified by more directed and in-depth research.
